# Increase in local protein concentration by field-inversion gel electrophoresis

**DOI:** 10.1186/1477-5956-5-18

**Published:** 2007-09-26

**Authors:** Henghang Tsai, Teck Yew Low, Steve Freeby, Aran Paulus, Kalpana Ramnarayanan, Chung-pui Paul Cheng, Hon-chiu Eastwood Leung

**Affiliations:** 1 Medical Proteomics and Bioanalysis Section, Genome Institute of Singapore, Singapore; 2 Bio-Rad Laboratories, Hercules, USA; 3 Department of Molecular and Cellular Biology, Department of Pediatrics, Baylor College of Medicine and Texas Children's Hospital, Houston, USA

## Abstract

**Background:**

Proteins that migrate through cross-linked polyacrylamide gels (PAGs) under the influence of a constant electric field experience negative factors, such as diffusion and non-specific trapping in the gel matrix. These negative factors reduce protein concentrations within a defined gel volume with increasing migration distance and, therefore, decrease protein separation efficiency. Enhancement of protein separation efficiency was investigated by implementing pulsed field-inversion gel electrophoresis (FIGE).

**Results:**

Separation of model protein species and large protein complexes was compared between FIGE and constant field electrophoresis (CFE) in different percentages of PAGs. Band intensities of proteins in FIGE with appropriate ratios of forward and backward pulse times were superior to CFE despite longer running times. These results revealed an increase in band intensity per defined gel volume. A biphasic protein relative mobility shift was observed in percentages of PAGs up to 14%. However, the effect of FIGE on protein separation was stochastic at higher PAG percentage. Rat liver lysates subjected to FIGE in the second-dimension separation of two-dimensional polyarcylamide gel electrophoresis (2D PAGE) showed a 20% increase in the number of discernible spots compared with CFE. Nine common spots from both FIGE and CFE were selected for peptide sequencing by mass spectrometry (MS), which revealed higher final ion scores of all nine protein spots from FIGE. Native protein complexes ranging from 800 kDa to larger than 2000 kDa became apparent using FIGE compared with CFE.

**Conclusion:**

The present investigation suggests that FIGE under appropriate conditions improves protein separation efficiency during PAGE as a result of increased local protein concentration. FIGE can be implemented with minimal additional instrumentation in any laboratory setting. Despite the tradeoff of longer running times, FIGE can be a powerful protein separation tool.

## Background

Sodium dodecyl sulfate-polyacrylamide gel electrophoresis (SDS-PAGE) is an indispensable technique in protein separation. This technique has only changed marginally over the past three decades [1]. Despite its popularity, SDS-PAGE, as well as native PAGE for protein separation, suffers the basic limitation of band broadening by diffusion and trapping of biomolecules in gel matrices. Nevertheless, protein separation by SDS-PAGE interfaced with mass spectrometry (MS) has currently emerged as the method of choice in the forefront of proteomics. Thus, new tools for upstream gel electrophoresis that can improve protein separation efficiency and subsequent detection will possibly lead to new discoveries in downstream processes.

Pulsed-field gel electrophoresis (PFGE) is an elegantly simple and universally accepted technique for the separation of large DNA molecules [2]. Several variant forms of PFGE with different experimental electrophoretic configurations now exist [3-5]. One variant is the orthogonal-field-alternation gel electrophoresis (OFAGE) technique [3]. Another variant is the field-inversion gel electrophoresis (FIGE) in which net forward molecular migration is achieved by either employing a longer net forward field time or a higher forward field strength compared with the reverse direction [4]. Application of FIGE in the separation of large DNA molecules was first reported two decades ago [4]. Among the various pulsed-field gel electrophoresis techniques, FIGE is likely the easiest to perform with minimal special equipment that generates a highly uniform electric field across the gel [4]. Both methods have been optimized to maximize efficiency (band width in the dimension of separation) and selectivity (distance between the center of two bands) for large DNA molecules. Systematic investigations of FIGE on the separation of DNA molecules in agarose gels were previously reported [6-10]. However, published studies of the use of FIGE for protein separation are still lacking.

Attempts were made to enhance the separation of proteins by means of PFGE [11]. Subsequent applications of PFGE were used to resolve either specific model protein species [12,13] or high molecular mass muscle myosin heavy chain isoforms [14]. However, these approaches were limited to the application of alternating cycles of on-and-off electric fields across a slab gel. This approach inevitably allowed diffusion to occur during the off times. Unwanted band broadening as a result of diffusion compromises general separation efficiency.

There are currently three models to explain molecular migration during PAGE: I) The Extended Ogston (EO) model assumes an overall sphere-like conformation for native or small proteins in which mobility is a function of available gel pores in a regular lattice fashion [15]. II) The reptation model assumes that molecules go through a rather disordered matrix, such as polyacrylamide [16], accounting for the snake-like movement of beads-on-a-string shaped polymers, such as protein-SDS complexes. These two models only apply to polypeptides within a certain molecular mass (MW) range at a given cross-linked polyacrylamide concentration. Any deviation from this linearity implies a change or transition in molecular shape (i.e., the radius) and net charge. III) The door-corridor (DC) model explains the behavior of polypeptides above a critical MW where the relative mobility of a protein becomes independent of the cross-linked polyacrylamide concentration [17]. Effective trapping of migrating molecules by the matrix predominates in this model and electrokinetic energy is required to overcome the trapping effect [17].

**Figure 1:**
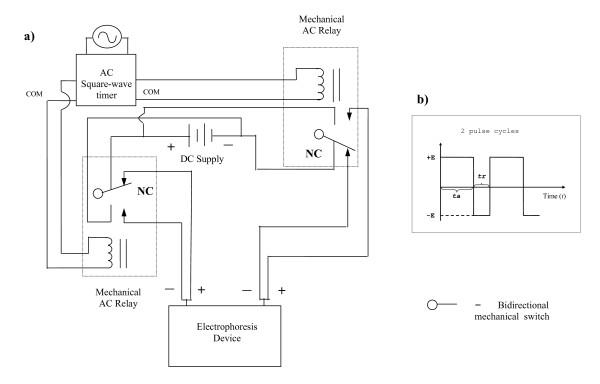
**Electric circuit of pulse generator and field diagram**. a) Electric circuit diagram for generating positive (+E) and negative (-E) square-wave electric fields during field inversion experiments. Direct current (DC) supply is from an external source. NC, normally closed switch; AC relay, alternate current relay; Com, common outlet. Inset b) shows a profile of the electric field during a typical FIGE experiment where + E = - E and *t*a (forward field time) is longer than *t*r (reverse field time).

We report here the engineering of a simple field-inversion device (Figure 1) and an initial study of protein separation efficiency using FIGE. The focus is to determine the ability of FIGE in the reduction of diffusion of a wide spectrum of protein species in a PAG matrix. The reduction of protein diffusion in polyacrylamide gel electrophoresis may lead to higher protein concentration per unit gel volume. The increased local protein concentration may therefore enhance tryptic peptide recovery in subsequent mass spectrometry analysis.

## Results

### The intensities of protein bands increase upon pulsing

**Figure 2:**
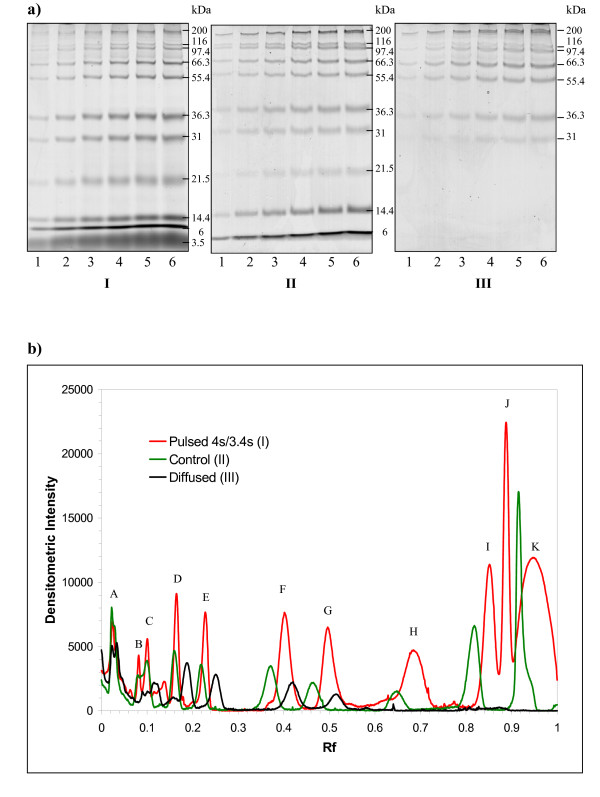
**Increased local concentrations of protein bands upon pulsing**. Protein band intensity analyses in FIGE (a I), CFE (a II), and CFE followed by resting within glass plates in room temperature for 12 hours (a III). Lanes 1 to 6 are 2 μL, 4 μL, 6 μL, 8 μL, 10 μL, and 12 μL of Mark12 protein standards, respectively, in a self-cast Bio-Rad 14% SDS-PAGE 1 mm × 7 cm gel followed by Coomassie blue staining. a I) Gel was run with a pulsed-field at (4 sec/3.4 sec) at 200 V for 13 hours, with an average buffer temperature of 30°C. A II) Gel was run at a constant field of 200 V for one hour and an average buffer temperature of 25°C. a III) Gel was run at a constant field of 200 V for one hour and left at rest for another 12 hours within the glass plates to permit diffusion prior to staining. b) Densitometry analysis of protein bands in the gels of the three conditions tested. Molecular mass was represented by alphabet A to K, where A = 200 kDa, B = 116.3 kDa, C = 97.4 kDa, D = 66.3 kDa, E = 55.4 kDa, F = 36.3 kDa, G = 31.0 kDa, H = 21.5 kDa, I = 14.4 kDa, J = 6.0 kDa, and K = unresolved 3.5/2.0 kDa bands, respectively. Migration distance relative to the dye front (R_f_) and intensity of bands from lane 6 of all three gels was densitometrically analyzed using Quantity One software. The graph results were the average of two independent experiments. The graph results were subsequently employed in the calculation of peak variance, σ^2^, in Table 1.

We investigated the effect of pulsing on the protein band intensity across a range of molecular masses; twelve protein species were separated by 14 % PAGE. The results showed that FIGE increased band intensities (Figure 2a I) compared with the CFE control (Figure 2a II). This notion was further investigated quantitatively using densitometry (Figure 2b). Protein peak heights were increased two-fold for protein molecular mass lower than 66 kDa. The enhancement of intensity for protein with molecular mass larger than 97 kDa was not obvious at this gel percentage and under these pulsing conditions. The increase in band intensities may be a result of reduced band diffusion and trapping of protein molecules in the gel matrix during migration. Diffusion became apparent when a control gel was run and allowed to rest for a further 12 hours within the glass plates at 20°C prior to staining (see Figure 2a III). The differences in band intensities were not caused by artifacts in staining and scanning as the parameters for staining and scanned were identical within the same set of experiments. Diffusion was not due to an increase in temperature as the temperature of gel during pulsing was 5°C higher than that of constant field controls. It was apparent that FIGE reduced band diffusion over a longer run time, since the run time required in this case was approximately 13-fold greater than that of the control. A general reduction in the full-width-half-maximum measure as a result of pulsing with respect to control was observed, suggesting that pulsing improved the overall efficiency of protein separation (Table 1). The improvement in separation efficiency becomes less obvious for relatively high molecular mass (≥ 200 kDa) and relatively low molecular mass (≤ 21.5 kDa) proteins. In summary, protein band intensity could be enhanced using FIGE.

**Table 1:** Peak variance (σ^2^) of proteins separated as in Figure 2a I by FIGE (Pulsing) and Figure 2a II by CFE (Control).

Peak^#^	A	B	C	D	E	F	G	H	I	J
MW (kDa)	200	116.3	97.4	66.3	55.4	36.5	31.0	21.5	14.4	6.0

σ^2 ^(Peak variance*, mm^2^)										

Pulsed	1.85 ± 0.07	0.41 ± 0.05	0.85 ± 0.12	0.87 ± 0.07	1.32 ± 0.15	4.51 ± 0.24	4.51 ± 0.38	8.51 ± 1.00	4.51 ± 0.15	1.52 ± 0.05
Control	1.62 ± 0.05**	U^†^	U	1.62 ± 0.15	2.63 ± 0.13	5.86 ± 0.28	5.86 ± 0.45	6.94 ± 0.42	4.51 ± 0.20	1.52 ± 0.11

^#^Peak label and shape can be found in Figure 2b

*Peak variance† (σ^2^) = (Full-width-half-maximum)^2^/5.54

** Results are the average of two experiments ± standard deviation

^†^U, peaks were not resolved and thus no peak variance numbers were available

### Altered protein relative mobility depend on pulse time settings and PAG percentages

**Figure 3:**
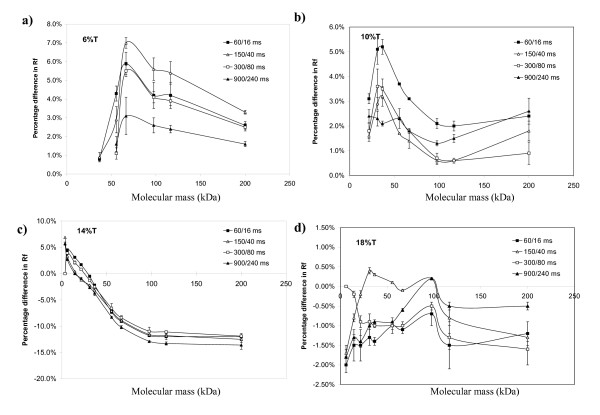
**Changes in relative mobility upon different pulsing conditions**. Comparison of changes in protein relative mobility between FIGE and CFE conditions in 6% (a), 10% (b), 14% (c) and 18% (d) cross-linked polyacrylamide concentration self-cast Bio-Rad SDS-PAG (1 mm × 7 cm). Different concentrations of polyacrylamide were casted in a mini-Protean 3 apparatus. Five microliters of Mark12 protein standards were used. Relative mobility was measured as a ratio of the migration distance of the target protein to that of the resolving front (% R_f_). The graphs were generated using Quantity One software. The y-axis denotes the percent differences of % R_f _in pulsed conditions compared to the CFE control. Each data point was the average of two separate experiments. All gels were run at 200 V with the average buffer temperature of 10°C. Positive values denote shorter migration distance and negative values denote longer migration distance with respect to CFE control. Error bar denotes the standard deviation of two separate experiments. Error bar cannot be showed if the range is smaller than the label.

We studied the effects of different ratios of forward pulse times (*ta*) and backward pulse times *(tr*) on protein *relative mobility *under different percentages of PAGs. Relative mobility was defined as the ratio of the migration distance of the target protein to the migration distance of the dye front (R_f_). The relative mobility was abbreviated as the percentage of R_f _(% R_f_). The % R_f _did not involve run time as it measured the position of the proteins when the dye front reached the same position regardless of the run time. Biphasic changes of % R_f _were observed at 6%, 10%, and 14% of PAGs (Figure 3a to 3c) under the tested pulsing conditions. More reduction in % R_f _was observed with protein size of 36 kDa to 66 kDa. The reduction decreased when protein species were more massive than 66 kDa. The apex (i.e., the molecular mass that showed the maximum difference in % R_f_) shifted to smaller molecular mass species when the gel percentage increased (Figure 3a, 3b, and 3c). Higher frequencies pulse cycles (*t*a/*t*r = 60/16 msec, 150/40 msec, and 300/80 msec) resulted in more obvious differences in relative mobility compared to low frequency pulse cycle (*t*a/*t*r = 900/240 msec) (Figure 3a to 3b). This change upon different pulse cycles was observed in the repeat run (Figure 3a and 3b). However, we did not see a simple linear relationship changes in relative mobility to gel concentration, pulse frequency, and protein size. We observed negative values in differences of % R_f _(Figure 3c and 3d) relative to CFE at high percentages of PAGs, suggesting that the target proteins revealed a longer migration distance. However, we did not observe a consistent pattern of altered relative mobility at 18 % PAG. The migration of proteins in such a high polyacrylamide concentration was apparently a stochastic process (Figure 3d). In summary, the results showed that the maximum difference in relative mobility was not a simple linear relationship with molecular mass, pulse cycles, and gel percentage.

### Pulsing during the second dimension separation enhances overall data output of 2D PAGE

**Figure 4:**
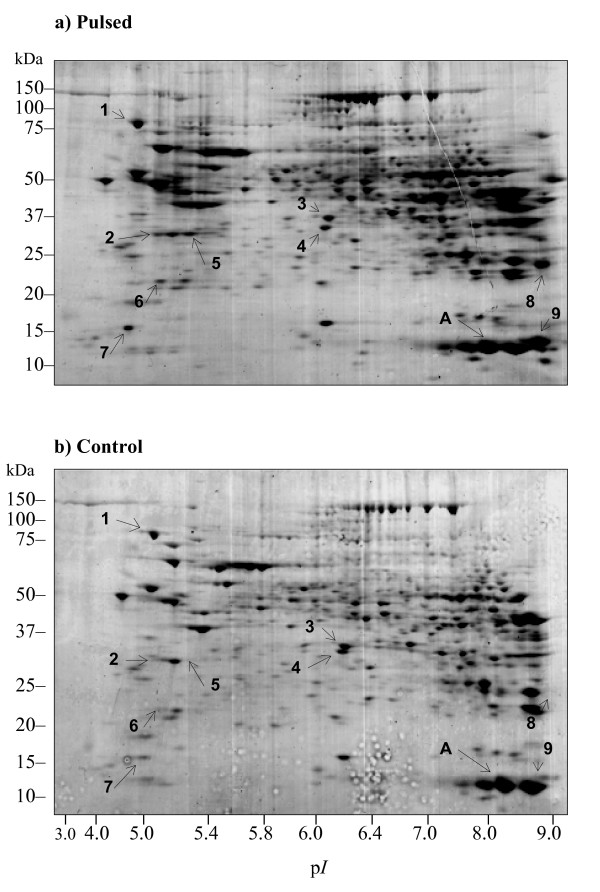
**Effects of FIGE on 2D PAGE analysis of rat liver lysates**. Comparison of 2D PAGE images of rat liver lysate under FIGE (a) and CFE (b) conditions. Each gel represents 100 μg of rat liver lysate separated by isoelectric focusing (IEF) using a non-linear pH 3–10 IPG strip in the first dimension and a Criterion precast SDS-10–20% PAG in the second dimension at room temperature. Control denotes CFE and pulsed denotes FIGE with a *t*a/*t*r of 400/106 msec in the second-dimension separation. Gels were stained with Coomassie blue. Spots selected for LC-MS/MS analysis are denoted by numbers, and A denotes the internal calibrator (see also Table 3). The internal calibrator is the internal controls for equivalent sample loading in both control and pulsed conditions.

We tested the applicability of FIGE in proteomics studies such as the 2D PAGE analysis of rat liver lysates with pulsing in the second dimension. The results were subsequently analyzed by image densitometry for spot number and intensity. Image analysis concluded that pulsing increased the overall number of spots by more than 20% as well as a general increase in spot intensity over CFE (Figure 4a and 4b). The total number of spots detected after CFE and FIGE were 477 and 611, respectively. In another set of experiments, gels were stained with Coomassie blue stain and the total number of detectable spots after CFE and FIGE were 190 and 240, respectively (data not shown). These results translated into a 26% increase in the total number of detectable spots.

We next studied the level of enhancement in protein spot intensity using densitometric analysis of selected spots. The enhancement of spot intensity ranged from 12 % to more than 300% on selected protein species (Table 2). We confirmed that this enhancement was not a result of sample loading or focusing error by including an internal control sample spot. Densitometry results of spot A (an internal control of a molecular mass standard protein) showed very similar densitometric values (31998.386 for control and 33593.516 for pulsing; Table 3); these results therefore allowed the dismissal of the observed pulsing effects as a consequence of handling error.

**Table 2:** Selective comparison of densitometric results from 2D PAGE analyses under FIGE versus CFE conditions

Spot number	Protein name	Pulsed (FIGE)	Control (CFE)	Percent Increase in Intensity (%)
		
		Densitometric intensity^#^		
1	Endoplasmin precursor	14414	8502	69
2	Regucalcin	11453	11006	4
3	Fructose-1,6-bisphosphatase	15358	13699	12.1
4	Malate dehydrogenase, cytoplasmic	14771	12522	17.9
5	Regucalcin Isoform	11266	*	N.A.
6	Lactoylglutathione lyase	8531	4603	85.3
7	Cytochrome b5	12585	5569	126
8	Glutathione S-transferase kappa1	18944	3813	396.8
9	Fatty acid-binding protein, liver	26493	6228	325.3
A	Internal calibrator	33593	31998	5

^#^Densitometric results are given as the average of the intensity per unit area. For similar spots under comparison, approximately the same area was obtained for the calculation of intensity. * denotes that the software failed to detect any differences with respect to backgroud noise. All results were corrected for background noise. Percent enhanced intensity is the percentage of increase in intensity using the intensity values of CFE as the denominator and the difference in intensity as the numerator. N.A., Not available.

**Table 3:** Selective comparison of MS results of protein spots identified from 2D PAGE under FIGE versus CFE conditions

Spot number^#^	Protein name	Mass (kDa)/p*I*	Pulsed (FIGE)		Control (CFE)	
			
			Score MASCOT	% Coverage	Score MASCOT	% Coverage
1	Endoplasmin precursor	92.8/4.74	535	21	143	13
2	Regucalcin	33.9/5.15	253	20	180	23
3	Fructose-1,6-bisphosphatase	37.2/6.18	457	27	358	28
4	Malate dehydrogenase, cytoplasmic	36.5/6.16	288	21	86	5
5	Regucalcin Isoform	33.9/5.20	493	30	*	*
6	Lactoylglutathione lyase	20.9/5.25	94	17	*	*
7	Cytochrome b5	15.1/4.96	74	41	*	*
8	Glutathione S-transferase kappa1	^25.7/8.97^	113	16	*	*
9	Fatty acid-binding protein, liver	14.3/8.59	367	25	*	*

^#^Protein spots 1–4 (Control) and 1–9 (Pulsed) as shown in Figure 6a were selected for LC-MS-MS analysis. * denotes indiscernible spots in control condition but discernible in pulsed conditions.

The spacing between spots 3 (37.2 kDa) and 4 (36.5 kDa) in the CFE control (Figure 4a) was much narrower than the spacing of matching spots in the pulsed condition (Figure 4b). We took into consideration that the migration distance of the dye front was the same for both cases. This observation suggested that FIGE might be used to separate proximal MW protein species or isoforms within a particular MW range under the influence of a given PAG concentration and pulse regime.

To confirm that the increase in spot intensity was not an artifact of pulsing, we further analyzed spots from identical positions between the pulsed and control gels with peptide sequencing using tandem mass spectrometry (MS). The MS results on nine selected protein spots revealed that pulsing increased the overall protein identification scores over control (Table 3). Weak intensity spots in CFE became apparent after pulsing (spots 5 to 9 in Figure 4a). The MS data allowed identification of these apparent spots with high ion scores (see Table 3 spots 5 to 9). These results confirmed the observation of increased band intensities as a consequence of increase in local protein concentrations of the same migrating protein species. In summary, pulsing in the second dimension enhanced local protein concentration during migration and, thus enhancing downstream processes such as mass spectrometry analysis.

### Native PAGE upon pulsing improves data output

**Figure 5:**
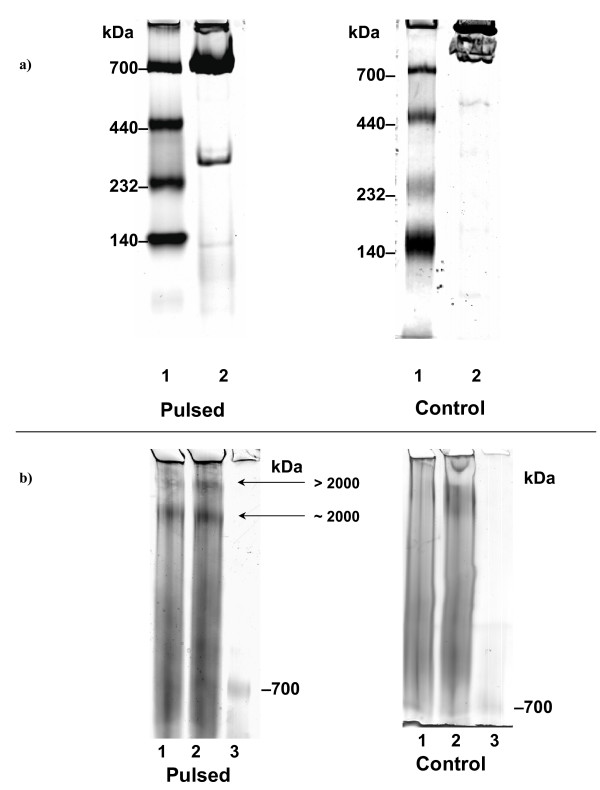
**Effects of FIGE on protein separation under native gel conditions**. Comparison of GroEL 14-mer complex (840 kDa) (a) or MCF-7 cell nuclear extract (b) in native PAG between CFE and FIGE conditions. FIGE was the left panel. CFE control was the right panel. The gels were 1 mm × 7 cm native 6% PAG casted in a mini-Protean 3 apparatus. Run time was 2 hrs 15 min in control condition. Pulsed condition run time was 5 hrs 30 min with a *t*a/*t*r of 400/100 msec. Lane 1, native MW markers (10 μg total); Lane 2, 10 μg purified *E. coli *GroEL native complex (14-mer, 840 kDa). The band at 300 kDa could be a minor cofactor associated with GroEL during purification. The gel was stained with Coomassie blue. Proteins were purposefully overloaded to best represent the effect of "detrapping" of native proteins under pulsing conditions. In panel b), MCF-7 nuclear extracted were run through native 2–5% PAG. The run time was 10 hrs for CFE control condition. Pulsed condition run time was 14 hrs with *ta*/t*r *of 900 msec/240 msec. Lane 1, 5.6 μg MCF-7 cell nuclear extract; Lane 2, 7.5 μg MCF-7 cell nuclear extract; Lane 3, native protein MW markers. The gels were silver stained.

We further explored the application of FIGE in the separation of protein complexes in native gel conditions. We discovered that the purified *E. coli *GroEL 14-mer complex (840 kDa) could effectively condense and migrate into the gel matrix under pulsing conditions of 900/240 msec (Figure 5a right panel). In contrast, the GroEL 14-mer complex migrated in a stochastic fashion under constant field conditions (see Figure 5a left panel). The difference was not due to an inappropriate buffer composition as the appearance of the GroEL 14-mer complex band was sharper than that in the CFE control. In addition, the protein bands of the MW protein markers were sharper after pulsing than the diffused bands in the control (see Figure 5a). Next, we tested a complex sample using MCF-7 cell line nuclear extract under native PAGE conditions. The results revealed that pulsing effectively condensed protein complexes greater than 2000 kDa in size, with a better resolution and band intensity (Figure 5b). In summary, FIGE might also be used to resolve very high molecular mass (MW > 1000 kDa) protein complexes by native PAGE.

## Discussion

We report here the first attempt to investigate certain common parameters on the separation of protein species by FIGE. These parameters include protein size, pulsing time ratios, pulsing frequencies, and cross-linked polyacrylamide percentage.

Despite longer running time and a higher running temperature, the protein species under FIGE conditions showed better separation efficiency than CFE. Higher intensity of protein bands or spots after pulsing could be explained by reduction of trapping of protein molecules by gel matrix along the direction of migration. When initial finite populations of polypeptide molecules comigrate in the direction of the electric field, a fraction of polypeptide molecules are continuously trapped along the gel matrix. FIGE relaxes and releases the trapped polypeptide molecules upon field reversal and thus renews the migration path. This field reversal increases the probability of the same polypeptide species to comigrate as a coalesced population. The detrapping hypothesis was in concordance with the observation in Figure 2a) and 2b) and Figure 5a) that protein intensity was consistently higher in pulsed condition despite the same amount of proteins were loaded. However, more massive proteins experience more constriction in migrating through the gel matrix and hence they are less likely to be detrapped upon pulsed condition. This hypothesis may be used to explain the similar protein intensity of more massive protein peaks A, B, and C in Figure 2b. Higher intensity could not be addressed by diffusion disentanglement process because it is dynamically time consuming [18,19], leading to diffusion to all directions from the origin. Therefore, one should expect lower local protein concentration by diffusion upon longer time or higher temperature. The consistent observation of enhanced protein intensity irrespective of running temperature suggests that the increased local concentration of protein was a genuine concentrating effect by FIGE.

Improved separation efficiency was also observed in the nonlinear focusing of DNA macromolecules according to the temporal ratchet hypothesis in which a short reverse pulse or spike reduced band broadening [18]. This implies that a similar operating mechanism may operate in the separation of protein molecules in PAGs. Furthermore, it was shown that asymmetric periodic electric fields led to the self-focusing of DNA within a gel [20]. This principle achieved a zero-integrated field electrophoresis phenomenon [18]. Similarly, every reversion of the electric field in FIGE generates a drift velocity. In the first case, where *t*a is longer than *t*r, the time-averaged drift velocity will be in the forward direction. At an intermediate point, where the forward and reverse drift velocities are equivalent and a zero drift velocity is attained, the molecules are focused in a condition termed *virtual trap *[20]. Intuitively, a greater focusing effect can be observed if the value of *t*a/*t*r is closer to 1. Our preliminary trial found that the best range of *ta*/*tr *lied between 1.07 and 3.75. The deviation from the best theoretical *ta*/*tr *ratio of 1 may be addressed by the fact that this process is in a non-equilibrium state. A dynamic interaction of proteins with fluxes of electrolytes in the buffer may add additional mobility driving force to the target protein molecule. A future study should involve the detailed investigation of the kinetics of electrolyte fluxes during pulsing.

Pulsing condition in general retards the relative mobility of extremely low mass proteins and accelerate the relative mobility of medium mass proteins (Figure 2b) under the right PAG concentration. In contrast, the relative mobility of extremely massive proteins remains unchanged. The shift in relative mobility cannot be simply explained by differences in thermodynamic energy generated during electrophoresis. The experimental temperature during the band intensity analysis was higher under pulsing conditions (Figure 2) while the temperature for generating Figure 3 data was controlled at 10°C. A consistent mobility shift was observed when pulsing was applied even under different temperatures, suggesting that temperature may not play a major role in the phenomenon.

We attempted to apply the three models to explain the altered relative mobility of polypeptides in FIGE. The EO model can be applied to the proteins with mass smaller than i) 66 kDa in 6% PAG concentration or ii) 36 kDa in 10% PAG concentration (Figure 3a and Figure 3b). The assumption of the EO model is that protein-SDS complexes are sphere-like structure. They migrate through relatively large pores in the gel matrix. Pulsing causes the protein molecules to reorientate in a periodic fashion. The process of reorientation leads to a reduction in relative mobility. As protein mass increases so does the reorientation time, resulting in a reduced relative mobility. Reptation model can be used to explain the migration of proteins with mass smaller than 100 kDa through the gel matrix at 14% PAG concentration (Figure 3c). In this model, the conformation of SDS-protein molecules may appear as a semi-chain like structure. Proteins adapt the most thermodynamically favorable conformations during migration through the gel matrix. The favorable conformation leads to an increase in relative mobility with increased detrapping. Apparently, the detrapping effect was plateaued for proteins with mass larger than 100 kDa because highly massive proteins (MWs higher than 100 kDa in 10 % PAG concentration) experience greater resistance from the relatively small pores in the gel matrix (Figure 3b). Their mobilities are constricted by gel matrix and therefore pulsing does not cause any significant changes in the relative mobility. A minimal decrease in the relative mobility may be the consequence of mostly localized reorientation under the given pulse regimes. At 18% PAG concentration, detrapping of polypeptides by FIGE is not realized. The average pore size of the gel is too small for the polypeptides to overcome local constriction even in the presence of pulsing. Polypeptides migrate through the gel according to the DC model in this high percentage gel condition. This observation is consistent with the behavior of DNA migration in previous study [21]. The relative mobility shift observed upon pulsing is more likely to be contributed by the interplay of the three protein migration models mentioned earlier. However, the inadequacy of all the models lies in the assumption of the properties of gel matrix. All models consider the gel to be either a rigid lattice of tubes, possibly allowing small variation in the size, or a collection of point obstacles in two dimensions. Study of molecular structure of polyacrylamide gel revealed that the pores exhibited certain degree of elasticity [22]. The change in properties of gel matrix under different pulsing conditions was not known. The elastic properties of gel matrix may contribute to a faster relative mobility of the protein molecules in one pulsing setting but a slower relative mobility in another pulsing setting.

FIGE suffers certain disadvantages when compared to other PFGE platform. Although simple in instrumental set up, FIGE suffers the limitation of lower resolving power than contour-clamped homogenous electric field (CHEF) electrophoresis. The resolution of very complex genomics structure can be achieved using two-dimensional CHEF electrophoresis [23]. This lower resolution of FIGE may be explained by the shorter migration route and suboptimal reorientation of migrating molecules. We have not studied in detail the separation of two protein species of similar molecular masses. In theory, the enhanced separation of two protein species with similar molecular masses can also be achieved with appropriate gel concentrations and pulsing conditions. It remains to be tested the lower limit of minimal molecular mass differences of two protein species that can be resolved using FIGE under optimized conditions. Another unaddressed general issue of PFGE linked to the increase in the width of band as seen transverse to the direction of migration. We observed a more prominent widening of spots at the left and right ends of 2D PAGE (Figure 4a and 4b). It was attributed by the difference in chain migration direction and the average direction of motion along the molecules [24]. Association of this difference with the pulsing condition needs further investigation. We can foresee that further improvements on limiting the transverse molecular motion can further improve the local concentration of protein species.

The present investigation tried to set the motion of establishing the potential application of FIGE in proteomics. If needed, further comprehensive analyses of upstream protein separation may be performed. We limited our investigation only to varying the *t*a/*t*r ratios under conditions of a constant electric field in different percentages of PAGs. It is, by no means, an exhaustive list of parameters tested. The present investigation suggests that FIGE is a *bona fide *protein separation technique that has the potential to complement existing CFE techniques in the area of SDS-PAGE, 2D PAGE, and native PAGE. FIGE aids in the reduction of thermo-diffusion within gels. Furthermore, preliminary results also demonstrated that FIGE could enhance the recovery of proteins during electroelution (data not shown). In future perspective, whether it will enjoy widespread acceptance as its counterpart in DNA analysis will depend on continued improvements and optimizations of different parameters. This further development requires parallel exploitation of experimental and computational modeling approaches to understanding FIGE in proteomics and eventually lead to the advent of new applications.

## Conclusion

FIGE enhances protein separation by improving local protein concentrations during SDS-PAGE or under native gel conditions. The increased local protein concentration thus improves the observable intensity of protein species in PAGs and also improves the success rate of downstream peptide sequencing using MS. Taken together, FIGE can be used to complement constant field gel electrophoresis for better protein separation and detection.

## Methods

### Instrumentation

A schematic representation of the pulsing circuitry in conjunction with an external electrophoresis unit is provided in Figure 1. A picture of the prototype can be found in supplementary data (see additional file 1). Forward and reverse switching of the electric field supply to the gel was achieved by interfacing the voltage supply (Power Pac 1000 power supply, Bio-Rad, Hercules, CA) with an 240 V alternate current (AC) relay (MY2, OMRON, Japan) that could handle a current of 5 Amp. The rate of the forward and reverse switching was controlled by a MD4E-AP programmable 110–240 V AC switching device (Fuji Electric, Japan) that was able to deliver pulses as short as one msec. Demonstration of the different settings can be found in additional file 2 and additional file 4. The performance of this simple instrumentation in regard to square wave form, amplitude, length, and stability of the pulses was checked and ascertained by a Tektronix oscilloscope model TDS 1000 (see additional file3 and see additional file 5).

### One-dimensional (1D) PAGE

We adopted a symmetric voltage method by varying both the forward and reverse pulse lengths in different percentages of cross-linked polyacrylamide. FIGE is herein referred to as pulsing and constant field electrophoresis (CFE) is herein referred to as control. Protein standards (2 μL, 4 μL, 6 μL, 8 μL, 10 μL, and 12 μL) from 2.5 kDa to 200 kDa (Mark12, Invitrogen Inc., Carlsbad, CA) were used as model proteins. A mini-PROTEAN 3 cell system (Bio-Rad Laboratories Inc., Hercules, CA) was used for the self-cast gel. The Criterion cell system (Bio-Rad Laboratories Inc., Hercules, CA) was employed for pre-cast gels used in the second dimension during 2D PAGE. Working solutions for gel casting were prepared from a 30% (w/v) acrylamide stock solution with 37.5:1 and 29:1 ratios of acrylamide to N, N'-methylene-bis-acrylamide for SDS-PAGE and native-PAGE, respectively. Percentages of slab gels used for protein separation were selected based on the sizes of the different proteins under investigation with reference to the migration chart for the protein standards obtained from the vendor (Bio-Rad Laboratories Inc., Hercules, CA). Final compositions of working solutions for the separating gel were based on the Laemmli buffer system [1]. A 1 × SDS running buffer or tank buffer (25 mM Tris, 192 mM glycine, 0.1% SDS, pH 8.3) was used with the Multitemp temperature control (GE Healthcare, Piscataway, NJ) at 10°C except as otherwise mentioned. All samples were mixed with 4 × sample loading buffer [0.24 M Tris- HCL, pH 6.8, 2% SDS, 3 mM bromophenol blue, 50.4% glycerol, 0.4 M dithiothreitol (DTT)] at a final concentration of 1 ×. All samples were heated to a temperature of 95°C for 1 minute prior to loading. All gels were run at 200 V until the dye-front reached the end of the gel. The temperature of the running buffer bathing the gel was taken at the beginning and at the end of each run. Subsequently, plots of the relative mobilities from the dye front (R_f_) of these proteins under different pulsing methods (different ratios of forward pulse times, *t*a, to different reverse pulse times, *t*r) and different percentages of cross-linked polyacrylamide were examined. Based on a series of trial and error experiments (data not shown) coupled with theoretical calculations [25], the *t*a/*t*r ratios tested were between 1.07 and 3.75. Gels were stained with colloidal Coomassie blue (Simply Blue, Invitrogen Inc., Carlsbad, CA) according to the vendor's instructions and subsequently scanned using the Labscan system (GE Healthcare, Piscataway, NJ) at a resolution of 600 dpi.

In the case of native PAGE, SDS was omitted from the working solution as described before [26]. *Escherichia coli *chaperonin GroEL was purified as previously described [27] and samples were stored at -86°C until use. Native 2–5% polyacrylamide minigels were cast using the Bio-Rad mini-Protean 3 multi-casting chamber (Bio-Rad Laboratories Inc., Hercules, CA) and gradient former (Model 485, Bio-Rad Laboratories Inc., Hercules, CA). Treatment of samples prior to loading was similar to those for SDS-PAGE except that SDS, DTT, and heating were omitted.

### Two-dimensional (2D) PAGE

Mouse liver protein lysate was prepared as described previously [28]. Protein concentrations were measured using the MicroBCA method (Pierce, Rockford, IL) with bovine serum albumin as a standard. Typically 100 μg of liver sample were resuspended in a final volume of 200 μL for the 11 cm ReadyStrip IPG strip, (immobilized non-linear pH 3–10 gradient from Bio-Rad) with rehydration solution (8 M urea, 2 M thiourea, 1% w/v CHAPS, 20 mM DTT, and 0.5% v/v Pharmalyte 3–10). The strips were then covered with mineral oil. Rehydration and isoelectric focusing (IEF) were performed in an IPGphor apparatus (GE Healthcare, Piscataway, NJ) at 20°C. The strips were actively rehydrated for 12 hr at a maximum of 30 V and subsequently focused using the following four steps: 500 V for 500 Vhr, 3000 V for 6000 Vhr, 5000 V for 10 kVhr, and maintained at 8000 V for 60–100 kVhr. The IPG strips were equilibrated twice for 20 min after IEF with gentle shaking in 10 mL equilibration buffer (50 mM Tris-HCl, pH 6.8, 6 M urea, 30% glycerol, 2% (w/v) SDS, and trace bromophenol blue). Two percent (w/v) DTT (Sigma Inc., St Louis, MO) was added to the first equilibration step followed by the addition of 2.5% (w/v) iodoacetamide (Sigma Inc., St Louis, MO) into the second equilibration step. The IPG strips were placed on top of the SDS pre-cast gels and sealed with 0.5% (w/v) agarose in SDS electrophoresis buffer (25 mM Tris, 192 mM glycine, 0.1% (w/v) SDS) before the second-dimensional separation. Precision protein standards (Bio-Rad Laboratories Inc., Hercules, CA) were used as molecular mass markers. Criterion Tris-HCl precast gels contained 10–20% cross-linked polyacrylamide with 1.0 mm thickness and a 2D well for an 11-cm IPG strip (Bio-Rad Laboratories Inc., Hercules, CA). The voltage was kept constant at 200 V and the temperature was kept at 10°C. The run was stopped when the dye front reached the bottom of the gel. The *t*a*/t*r was set at 400/100 msec for FIGE in the second dimension. Gels were stained with Simply Blue (Invitrogen Inc., Carlsbad, CA) and were subsequently scanned using the Labscan system (GE Healthcare, Piscataway, NJ) at 600 dpi. SYPRO-Ruby (Bio-Rad Laboratories Inc., Hercules, CA)-stained gels were imaged with the molecular imager FX system from Bio-Rad (Bio-Rad Laboratories Inc., Hercules, CA).

### Image and data analyses

Scanned 2D gel images were analyzed using either PDQuest 2D gel analysis software (Bio-Rad Laboratories Inc., Hercules, CA) or Z3 2D analysis software (Compugen, Israel). Scanned 1D gel images were analyzed using Quantity One 1-D analysis software (Bio-Rad Laboratories Inc., Hercules, CA).

Separation efficiency (i.e., band width or peak width, *w*, in the direction of separation) was obtained from graphic output employing the Quantity One software. The full-width-half-maximum (FWHM) was measured on the Quantity One graphic output. Assuming a Gaussian distribution, peak variance, σ^2^, related to FWHM as;

σ^2 ^= (FWHM)^2^/5.54

### Protein Identification

Protein bands or spots of interest on gels were excised and cut into 1- to 2-mm^2 ^pieces. All gel pieces were washed in ultra-pure water and were subsequently subjected to in-gel trypsin digestion [29,30]. The resulting peptides from each sample were extracted from the gel pieces twice with 50 μL of 50% (v/v) acetonitrile/0.1 % (v/v) trifluoroacetic acid and the supernatants were pooled. The extracted peptides were dried in a Speed-Vac prior to dissolution in 5% formic acid for LC-MS/MS analysis.

Typically 20–40 μL of each digest was applied to an Agilent 1100 series Nano-HPLC coupled to an Agilent LC/MSD XCT Plus ion trap mass spectrometer. Briefly, peptides were trapped on a Zorbax 300SB-C18 enrichment column (Agilent, Germany) before eluted onto a column packed with 5-μm Magic C18 particles (Michrom BioResources, Auburn, CA). The C18 column output led to an integrated, 15-μm orifice, fritted nanospray source (Agilent Technologies, Inc. Santa Clara, CA). The Nano-LC was operated in micro mode at a flow rate of 300 nL/min. The gradient was maintained at 5% buffer B (0.1% formic acid in acetonitrile) for 2 minutes before increasing to 40% buffer B at 40 minutes. The gradient was further increased to 60% B at 45 minutes, 95% B at 50 minutes, and maintained for 3 minute before decreasing to 5% B for another 2 minutes for equilibration. The MS was operated at a spray voltage of 1.5 kV, with dry gas (200°C) flowing at 2.0 L/min. MS/MS was performed in a data-dependent manner in that three of the most intense peaks from each MS spectrum were selected for subsequent MS/MS analysis. Active exclusion was activated after 1 spectrum and released after 60 seconds.

The mass spectra were later processed using Agilent Data Analysis software. Compounds were first created with selection criteria of a signal/noise threshold of 5 and a relative intensity and area threshold of 10%. The spectra were subsequently deconvoluted and exported as MASCOT generic files for searching the SwissProt database via an in-house MASCOT server. The taxonomical criterion was set to metazoan (animals) with a maximum of 2 missed cleavages using trypsin. Carbaminomethylation was set as the only fixed modification and tolerance was set to 2.0 Da and 0.8 for peptides and MS/MS values, respectively.

## Abbreviations

The **abbreviations **used are: FIGE, field-inversion gel electrophoresis; PFGE, pulsed-field gel electrophoresis; 2D PAGE, two-dimensional polyacrylamide gel electrophoresis; *t*a, forward pulse time; *t*r reverse pulse time; MW, molecular mass; PAG, polyacrylamide gel; CFE, constant-field electrophoresis; EO, Extended Ogston; σ^2^, peak variance; RM, relative mobility.

## Competing interests

The author(s) declare that they have no competing interests.

## Authors' contributions

HHT consolidated the study design, engineered the electronic field inversion device, performed most of the electrophoresis experiments, analyzed most of the results, and drafted the manuscript. TYL participated in rat liver lysate preparation and LC/MS/MS analysis of protein spots. SF and AP analyzed the Criterion gel results. KR carried out GroEL protein purification. CPC participated in 2D PAGE. HCEL formulated the study design, analyzed part of the results, and finalized the manuscript. All authors have read and approved the final manuscript.

## Supplementary Material

Additional file 1 The physical setup of the field-inversion device. Figure S1 shows the links of the pulsed-inversion device from the power pack to the gel tank. Click here for file

Additional file 2 The timer screen when the pulsed-inversion device was set at a pulse frequency of *t*a/*t*r = 80/40 msec. A movie showes the real-time readout of the changes of voltage output on the timer of the pulsed-inversion device. Click here for file

Additional file 3 The oscilloscope screen when the pulsed-inversion device was set at a pulse frequency of *t*a/*t*r = 80/40 msec. Description: A movie shows the real-time readout from the Tektronix oscilloscope model TDS 1000 when the pulsed-inversion device was set at a *ta*/*t*r = 80/40 msec. Click here for file

Additional file 4 The timer screen when the pulsed-inversion device was set at a pulse frequency of *t*a/*t*r = 160/40 msec. A movie showes the real-time readout of the changes of voltage output on the timer of the pulsed-inversion device when the device was set at a *t*a/*t*r = 160/40 msec. Click here for file

Additional file 5 The oscilloscope screen when the pulsed-inversion device was set at a pulse frequency of *t*a/*t*r = 160/40 msec. A movie shows the real-time readout from the Tektronix oscilloscope model TDS 1000 when the pulsed-inversion device was set at a *ta*/*t*r = 160/40 msec. Click here for file
