# Supplementary figures and images for: Increase in local protein concentration by field-inversion gel electrophoresis
Source: Proteome Sci. 2007 Sep 26;5:18. doi: 10.1186/1477-5956-5-18 (PMC2211458; doi:10.1186/1477-5956-5-18)

# Setup of Differential Field-Inversion Gel Electrophoresis

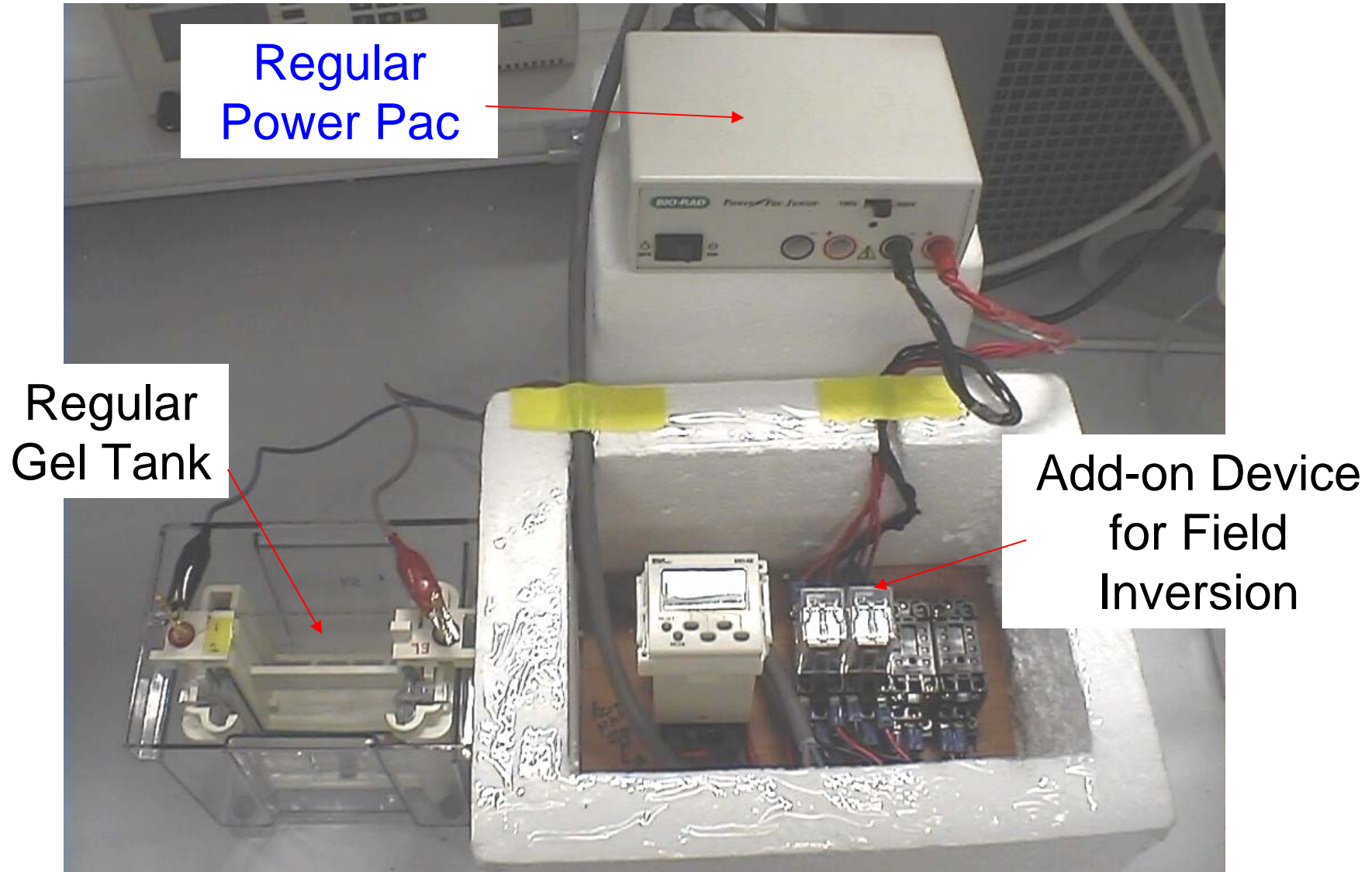

Supplement: Additional file 1 — The physical setup of the field-inversion device. Figure S1 shows the links of the pulsed-inversion device from the power pack to the gel tank. [file 1477-5956-5-18-S1.pdf]
